# Effectively suppressed reflected photonic spin Hall effect

**DOI:** 10.1515/nanoph-2025-0089

**Published:** 2025-03-26

**Authors:** Lijuan Sheng, Zixiao Xu, Yong Cao, Yawei Tan, Xiaohui Ling, Xinxing Zhou

**Affiliations:** College of Physics and Electronic Engineering, 12573 Hengyang Normal University, Hengyang 421002, China; Key Laboratory of Low-Dimensional Quantum Structures and Quantum Control of Ministry of Education, School of Physics and Electronics, Hunan Normal University, Changsha 410081, China; Key Laboratory of Physics and Devices in Post-Moore Era, College of Hunan Province, Changsha 410081, China; Institute of Interdisciplinary Studies, Hunan Normal University, Changsha 410081, China

**Keywords:** photonic spin Hall effect, effectively suppressed photonic spin Hall effect, reflective spin switches

## Abstract

The photonic spin Hall effect can engender transverse spatial and angular displacements in both transmission and reflection, with significant applications in optical imaging, edge detection, and the development of spin-based nanophotonic devices. While previous research has focused on enhancing the photonic spin Hall effect, suppression can be beneficial for photonic spin-switching, offering advantages such as increased speed and sensitivity in nanophotonic devices. In this study, we establish a quantitative correlation between the reflection coefficient and the transverse spatial and angular displacements of reflected light, as induced by the photonic spin Hall effect, grounded in electromagnetic theory. We find that the transverse spatial displacement of reflected light can be eliminated under the condition *r*_*pp*_ = −*r*_*ss*_, where *r* denotes the reflection coefficient, and the first (second) superscript denotes the polarization of the reflected (incident) light. This condition applies to light with arbitrary polarization states, at arbitrary incident angles, and is independent of wavelength and beam waist. A similar outcome is obtained for the transverse angular displacement of the reflected light. Such distinctive displacements are attainable through the use of an electromagnetic interface that satisfies the reflected condition *r*_*pp*_ = −*r*_*ss*_. Additionally, we provide a succinct overview of the methodologies for constructing reflective spin switches.

## 1 Introduction

The photonic spin Hall effect (PSHE) represents a quintessential phenomenon in the realm of light–matter interaction, rooted in the intricate interplay of spin and orbital angular momentum [1], [2], [3], [4], [5], [6]. A salient characteristic of PSHE is its remarkable sensitivity to the parameters of optical interfaces. Consequently, a suite of measurement methodologies has been devised leveraging the PSHE, encompassing techniques for the detection of chiral optical signals [7], [8], the characterization of two-dimensional material properties [9], [10], [11], [12], [13], and the assessment of magneto-optical parameters [14], [15], [16], [17]. Beyond facilitating the measurement of optical interface information, the PSHE has also demonstrated its utility in optical imaging [18], [19], edge detection [20], [21], [22], [23], and the innovation of spin photonics devices [24], [25], [26], [27], [28], [29], such as spin meta-lenses [24], [25], [26] and spin routers [27], [28]. Thus, the PSHE is pivotal across a multitude of scientific disciplines, including nano-photonics [30], [31], plasma science [32], [33], materials science [34], [35], and biomedical science [36], [37], and is actively expanding the horizons within these domains.

In previous studies, the enhancement of the PSHE has been commonly investigated [9], [10], [11], [12], [13], [14], [15], [16], [17], [18], [19], which is beneficial for detecting the physical properties of the interface and developing related applications. Conversely, the suppression of PSHE can also be advantageous, particularly in the context of spin-switching applications [38], [39], [40]. Compared to conventional switching mechanisms, the proposed system offers advantage such as increased speed, heightened sensitivity, and the capacity for multiplexing. In 2023, the exploration of the exotic PSHE at chiral interfaces was introduced [41]. It was demonstrated that when the Fresnel coefficients adhere to a particular relationship, the spin-induced transverse spatial displacement of transmitted light remains consistently null, paving the way for the development of spin-switching devices. The suppression can be beneficial for photonic spin-switching, offering advantages such as increased speed and sensitivity in nanophotonic devices. However, the effectively suppressed reflected PSHE remains obscure for further exploration.

In this study, we draw upon the electromagnetic theory of light to establish a relationship between the reflected transverse spatial and angular displacements engendered by the PSHE and the reflection coefficient. Through our analysis, we discern that these displacements can be effectively suppressed under specific conditions. Notably, when the interface satisfies the condition of *r*_*pp*_ = −*r*_*ss*_, both the reflected transverse spatial and angular displacements disappear significantly. Here, *r* denotes the reflection coefficient, with the first (second) superscript indicating the polarization of the reflected (incident) light. Achieving the aforementioned reflection condition is feasible, for instance, by utilizing an electromagnetic interface with electric and magnetic surface conductivities. Furthermore, the construction of reflective spin switches is also addressed. Our findings introduce new degrees of freedom for manipulating photonic spins and hold potential for applications within the domain of all-optical spin switches.

**Figure 1:**
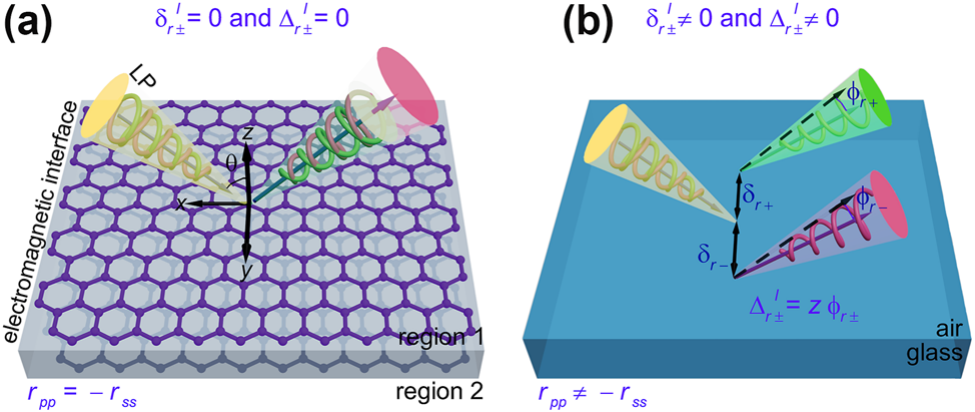
Illustration of the effectively suppressed PSHE of the reflected light. (a) A schematic representation of the effectively suppressed reflected PSHE at a judiciously designed interface (e.g., an electromagnetic interface) adhering to the condition *r*_*pp*_ = −*r*_*ss*_, where *LP* denotes linear polarization. (b) A schematic of reflected PSHE at a non-magnetic interface (e.g., the air-glass interface) with *r*_*pp*_ ≠ −*r*_*ss*_. In this context, the superscript *l* associated with transverse displacements indicates the linear polarization of incident light, while the subscript (namely *p* and *s*) of the reflection coefficient represents the polarization state of the incident light.

To quantitatively elucidate the PSHE, we now proceed to delineate the induced reflected spatial and angular displacements at a planar interface (refer to Figure 1(a) and (b)) for a Gaussian beam with arbitrary linear polarizations (e.g. ${\left(\begin{array}{cc} \cos\beta & \sin\beta \end{array}\right)}^{T}$ ) incident upon it. The angular spectrum of the incident Gaussian beam can be mathematically expressed as ${\bar{E}}_{i}=\left(\cos\beta {\hat{x}}_{i}+\sin\beta {\hat{y}}_{i}\right)\frac{w}{\sqrt{2\pi }}{\text{e}}^{-w^{2}\left(k_{ix}^{2}+k_{iy}^{2}\right)/4}$ , where *β* and *w* denote the polarization angle and the beam waist of the incident light, respectively, and $k_{ix}\left(k_{iy}\right)$ represents the wave vector component along the ${\hat{x}}_{i}\left({\hat{y}}_{i}\right)$ direction within the local coordinate system *x*_*i*_ *y*_*i*_ *z*_*i*_. Through coordinate transformation, we obtain the transformation matrix from the incident central angular spectrum to the incident arbitrary angular spectrum. By incorporating the Fresnel coefficient matrix $F_{r}=\left(\begin{array}{cc} r_{pp} & r_{ps}\\ r_{sp} & r_{ss} \end{array}\right)$ and the transformation matrix from the reflected arbitrary angular spectrum to the reflected central angular spectrum, the reflected central angular spectrum ${\bar{E}}_{r}$ can be derived [5], [6]. Specifically, the reflected center angular spectrum for the left-handed/right-handed circularly polarized component is given by ${\bar{E}}_{r\pm }=\left\{r_{pp}\cos\beta +r_{ps}\sin\beta \pm i\left(r_{ss}\sin\beta +r_{sp}\cos\beta \right)+\right.\left[\left(r_{pp}+r_{ss}\right)\left(\sin\beta \mp i\cos\beta \right)+\left(r_{sp}-r_{ps}\right)\left(\cos\beta \pm i\sin\beta \right)\right]\left.k_{ry}\cot\theta /k\right\}\frac{w}{2\sqrt{\pi }}e^{-w^{2}\left(k_{ix}^{2}+k_{iy}^{2}\right)/4}$ , where the subscripts + or – denote the left-handed or right-handed circularly polarized component. In accordance with electromagnetic wave theory, the transverse spatial and angular displacements for the left-handed/right-handed circularly polarized component are described by the following equations [6], [42]:

(1) $$\delta_{r\pm }=\left\langle {\bar{E}}_{r\pm }\right|i\frac{\partial }{\partial k_{ry}}\left|{\bar{E}}_{r\pm }\right\rangle /\left\langle {\bar{E}}_{r\pm }\left|{\bar{E}}_{r\pm }\right.\right\rangle$$

(2) $$\Delta_{r\pm }=\frac{z}{n_{\text{out}}k}\left\langle {\bar{E}}_{r\pm }\right|k_{ry}\left|{\bar{E}}_{r\pm }\right\rangle /\left\langle {\bar{E}}_{r\pm }\left|{\bar{E}}_{r\pm }\right.\right\rangle$$

where *n*_out_ represents the refractive index of the outgoing medium, *z* signifies the propagation distance, *k* = 2*π*/*λ* with *λ* being the wavelength of light in free space, and *k*_*ry*_ is the wave vector component along the ${\hat{y}}_{r}$ direction in the local coordinate system *x*_*r*_ *y*_*r*_ *z*_*r*_.

## 2 Reflected transverse spatial displacement

To generalize, we consider the scenario where cross-Fresnel coefficients are absent, namely $r_{pp}=\left|r_{pp0}\right|{\text{e}}^{\text{i}\varphi_{r_{pp0}}}-\frac{k_{rx}}{k}\left|r_{pp1}\right|{\text{e}}^{\text{i}\varphi_{r_{pp1}}}$ , $r_{ss}=\left|r_{ss0}\right|{\text{e}}^{\text{i}\varphi_{r_{ss0}}}-\frac{k_{rx}}{k}\left|r_{ss1}\right|{\text{e}}^{\text{i}\varphi_{r_{ss1}}}$ , and *r*_*ps*_ = *r*_*sp*_ = 0, where $\left|r_{\mathrm{pp}0}\right|$ (or $\left|r_{\mathrm{ss}0}\right|$ ) and $\varphi_{r_{\mathrm{pp}0}}$ (or $\varphi_{r_{\mathrm{ss}0}}$ ) represent the amplitude and phase of the zero-order term of the reflection coefficient *r*_*pp*_ (or *r*_*ss*_), respectively. Correspondingly, $\left|r_{\mathrm{pp}1}\right|$ (or $\left|r_{\mathrm{ss}1}\right|$ ) and $\varphi_{r_{\mathrm{pp}1}}$ (or $\varphi_{r_{\mathrm{ss}1}}$ ) correspond to the first-order terms. After a rigorous derivation, the reflected transverse spatial displacement for the left- and right-handed circular polarization components can be articulated as follows:

(3) $$\delta_{r\pm }^{l}=\mp \frac{kw^{2}\cot\theta \left(A+B\right)}{C+D\pm F}$$

where in $A=k^{2}w^{2}\left\{{\left|r_{\mathrm{pp}0}\right|}^{2}\cos^{2}\beta +{\left|r_{\mathrm{ss}0}\right|}^{2}\sin^{2}\beta +\left|r_{\mathrm{pp}0}\right|\right.\left.\left|r_{\mathrm{ss}0}\right|\left[\cos\left(\varphi_{r_{\mathrm{ss}0}}-\varphi_{r_{\mathrm{pp}0}}\right)\pm \sin2\beta \sin\left(\varphi_{r_{\mathrm{ss}0}}-\varphi_{r_{\mathrm{pp}0}}\right)\right]\right\}$ , $B={\left|r_{\mathrm{pp}1}\right|}^{2}\cos^{2}\beta +{\left|r_{\mathrm{ss}1}\right|}^{2}\sin^{2}\beta +\left|r_{\mathrm{ss}1}\right|\left|r_{\mathrm{pp}1}\right|\left[\cos\left(\varphi_{r_{\mathrm{pp}1}}-\varphi_{r_{\mathrm{ss}1}}\right)\mp \sin2\beta \sin\left(\varphi_{r_{\mathrm{pp}1}}-\varphi_{r_{\mathrm{ss}1}}\right)\right]$ , $C=\cos^{2}\beta \left\{k^{2}w^{2}\left(k^{2}w^{2}{\left|r_{\mathrm{pp}0}\right|}^{2}+{\left|r_{\mathrm{pp}1}\right|}^{2}\right)+\right.\left.\left[k^{2}w^{2}\left({\left|r_{\mathrm{pp}0}\right|}^{2}+{\left|r_{\mathrm{ss}0}\right|}^{2}+2\left|r_{\mathrm{pp}0}\right|\left|r_{\mathrm{ss}0}\right|\cos\left(\varphi_{r_{\mathrm{ss}0}}-\varphi_{r_{\mathrm{pp}0}}\right)\right)+\right.\right.\left.\left.{\left|r_{\mathrm{ss}1}\right|}^{2}+{\left|r_{\mathrm{pp}1}\right|}^{2}+2\left|r_{\mathrm{pp}1}\right|\left|r_{\mathrm{ss}1}\right|\cos\left(\varphi_{r_{\mathrm{pp}1}}-\varphi_{r_{\mathrm{ss}1}}\right)\right]\cot^{2}\theta \right\}$ , $D=\sin^{2}\beta \left\{k^{2}w^{2}\left(k^{2}w^{2}{\left|r_{\mathrm{ss}0}\right|}^{2}+{\left|r_{\mathrm{ss}1}\right|}^{2}\right)+\right.\left.\left[k^{2}w^{2}\left({\left|r_{\mathrm{pp}0}\right|}^{2}+{\left|r_{\mathrm{ss}0}\right|}^{2}+2\left|r_{\mathrm{pp}0}\right|\left|r_{\mathrm{ss}0}\right|\cos\left(\varphi_{r_{\mathrm{ss}0}}-\varphi_{r_{\mathrm{pp}0}}\right)\right)+\right.\right.\left.\left.{\left|r_{\mathrm{ss}1}\right|}^{2}+{\left|r_{\mathrm{pp}1}\right|}^{2}+2\left|r_{\mathrm{ss}1}\right|\left|r_{\mathrm{pp}1}\right|\cos\left(\varphi_{r_{\mathrm{pp}1}}-\varphi_{r_{\mathrm{ss}1}}\right)\right]\cot^{2}\theta \right\}$ , and $F=k^{2}w^{2}\sin2\beta \left[k^{2}w^{2}\left|r_{\mathrm{pp}0}\right|\left|r_{\mathrm{ss}0}\right|\sin\left(\varphi_{r_{\mathrm{ss}0}}-\varphi_{r_{\mathrm{pp}0}}\right)-\right.\left.\left|r_{\mathrm{ss}1}\right|\left|r_{\mathrm{pp}1}\right|\sin\left(\varphi_{r_{\mathrm{pp}1}}-\varphi_{r_{\mathrm{ss}1}}\right)\right]$ . The displacement is contingent not merely upon the polarization angle *β* and the incident angle *θ*, but also upon the wavelength *λ* and the beam waist *w* of the incident light. It is typically not-zero. Subsequently, we shall explore methodologies to eradicate the reflected transverse spatial displacement.

In the instance of purely *p*-polarized (transverse-magnetic, TM) incident light, the reflected transverse spatial displacement is simplified to

(4) $$\delta_{r\pm }^{p}=\mp \frac{kw^{2}\left(k^{2}w^{2}G_{0}+G_{1}\right)\cot\theta }{k^{2}w^{2}\left(k^{2}w^{2}{\left|r_{\mathrm{pp}0}\right|}^{2}+{\left|r_{\mathrm{pp}1}\right|}^{2}\right)+\left(k^{2}w^{2}J_{0}+J_{1}\right)\cot^{2}\theta }$$

where $G_{0}={\left|r_{\mathrm{pp}0}\right|}^{2}+\left|r_{\mathrm{pp}0}\right|\left|r_{\mathrm{ss}0}\right|\cos\left(\varphi_{r_{\mathrm{ss}0}}-\varphi_{r_{\mathrm{pp}0}}\right)$ , $G_{1}={\left|r_{\mathrm{pp}1}\right|}^{2}+\left|r_{\mathrm{pp}1}\right|\left|r_{\mathrm{ss}1}\right|\cos\left(\varphi_{r_{\mathrm{pp}1}}-\varphi_{r_{\mathrm{ss}1}}\right)$ , $J_{0}={\left|r_{\mathrm{pp}0}\right|}^{2}+{\left|r_{\mathrm{ss}0}\right|}^{2}+2\left|r_{\mathrm{pp}0}\right|\left|r_{\mathrm{ss}0}\right|\cos\left(\varphi_{r_{\mathrm{ss}0}}-\varphi_{r_{\mathrm{pp}0}}\right)$ , and $J_{1}={\left|r_{\mathrm{ss}1}\right|}^{2}+{\left|r_{\mathrm{pp}1}\right|}^{2}+2\left|r_{\mathrm{ss}1}\right|\left|r_{\mathrm{pp}1}\right|\cos\left(\varphi_{r_{\mathrm{pp}1}}-\varphi_{r_{\mathrm{ss}1}}\right)$ . To maintain simplicity, we shall consider a scenario where the reflection coefficient is not expanded using a Taylor series. When the reflection coefficient is purely real or imaginary at the interface (e.g., air-glass interface), such that $\varphi_{r_{\mathrm{pp}0}}=\varphi_{r_{\mathrm{ss}0}}=0$ , the displacement simplifies to $\delta_{r\pm }^{p}=\mp kw^{2}\left|r_{\mathrm{pp}0}\right|\left(\left|r_{\mathrm{pp}0}\right|+\left|r_{\mathrm{ss}0}\right|\right)\cot\theta /\left[k^{2}w^{2}{\left|r_{\mathrm{pp}0}\right|}^{2}+\right.\left.{\left(\left|r_{\mathrm{pp}0}\right|+\left|r_{\mathrm{ss}0}\right|\right)}^{2}\cot^{2}\theta \right]$ . Consequently, the conditions for the disappearance of displacement are $\left|r_{\mathrm{pp}0}\right|=0$ or $\left|r_{\mathrm{pp}0}\right|=-\left|r_{\mathrm{ss}0}\right|$ for *p*-polarized light. It is important to note that since *k* ^2^ *w* ^2^ is typically much larger than cot^2^ *θ*, equation (4) can be approximated as $\delta_{r\pm }^{p}\approx \mp \left({\left|r_{\mathrm{pp}0}\right|}^{2}+\left|r_{\mathrm{pp}0}\right|\left|r_{\mathrm{ss}0}\right|\right)\cot\theta /\left(kp^{2}\right)$ . For the case of $\left|r_{\mathrm{pp}0}\right|=0$ , both the numerator and denominator become zero, which implies that the reflected transverse spatial displacement at the Brewster angle is not null, as previously suggested in references [6], [9]. For the reflection condition $\left|r_{\mathrm{pp}0}\right|=-\left|r_{\mathrm{ss}0}\right|$ , it is applicable at arbitrary incident angles *θ*, irrespective of wavelength *λ* and beam waist *w*. Should the reflection coefficient be complex at the interface (e.g., metasurfaces), the reflected transverse spatial displacement is expressed as $\delta_{r\pm }^{p}=\mp kw^{2}\left[{\left|r_{\mathrm{pp}0}\right|}^{2}+\left|r_{\mathrm{pp}0}\right|\left|r_{\mathrm{ss}0}\right|\cos\left(\varphi_{r_{\mathrm{ss}0}}-\varphi_{r_{\mathrm{pp}0}}\right)\right]\cot\theta /\left(k^{2}w^{2}{\left|r_{\mathrm{pp}0}\right|}^{2}+J_{0}\cot^{2}\theta \right)$ . In this scenario, to effectively suppress displacement for *p*-polarized light, the requirement becomes $\left|r_{\mathrm{pp}0}\right|=0$ or $\left|r_{\mathrm{pp}0}\right|=-\left|r_{\mathrm{ss}0}\right|\cos\left(\varphi_{r_{\mathrm{ss}0}}-\varphi_{r_{\mathrm{pp}0}}\right)$ . Similar issues arise when $\left|r_{\mathrm{pp}0}\right|=0$ . For the reflection condition $\left|r_{\mathrm{pp}0}\right|=-\left|r_{\mathrm{ss}0}\right|\cos\left(\varphi_{r_{\mathrm{ss}0}}-\varphi_{r_{\mathrm{pp}0}}\right)$ , it is valid at arbitrary incident angles, encompassing variations in wavelength and beam waist. The phase of the reflection coefficient also plays a role in the emergence of this condition. For instance, if $\varphi_{r_{\mathrm{ss}0}}=\varphi_{r_{\mathrm{pp}0}}$ , the condition for the disappearance of the reflected transverse spatial displacement is $\left|r_{\mathrm{pp}0}\right|=-\left|r_{\mathrm{ss}0}\right|$ ; conversely, if $\varphi_{r_{\mathrm{ss}0}}=\varphi_{r_{\mathrm{pp}0}}\pm \pi$ , the condition becomes $\left|r_{\mathrm{pp}0}\right|=\left|r_{\mathrm{ss}0}\right|$ . Through analysis, it becomes evident that irrespective of the interface (air-glass interface or metasurface, etc.), the condition for effectively suppressing the reflected transverse displacement for incident pure *p*-polarized light is

(5) $$r_{pp}=-r_{ss}$$

for the incident pure *p*-polarized light.

Upon incidence of purely *s*-polarized (transverse-electric, TE) light, the reflected transverse spatial displacement is simplified to

(6) $$\delta_{r\pm }^{s}=\mp \frac{kw^{2}\left(k^{2}w^{2}K_{0}+K_{1}\right)\cot\theta }{k^{2}w^{2}\left(k^{2}w^{2}{\left|r_{\mathrm{ss}0}\right|}^{2}+{\left|r_{\mathrm{ss}1}\right|}^{2}\right)+\left(k^{2}w^{2}J_{0}+J_{1}\right)\cot^{2}\theta }$$

among other $K_{0}={\left|r_{\mathrm{ss}0}\right|}^{2}+\left|r_{\mathrm{pp}0}\right|\left|r_{\mathrm{ss}0}\right|\cos\left(\varphi_{r_{\mathrm{ss}0}}-\varphi_{r_{\mathrm{pp}0}}\right)$ , and $K_{1}={\left|r_{\mathrm{ss}1}\right|}^{2}+\left|r_{\mathrm{ss}1}\right|\left|r_{\mathrm{pp}1}\right|\cos\left(\varphi_{r_{\mathrm{pp}1}}-\varphi_{r_{\mathrm{ss}1}}\right)$ . For the simplified case, if the reflection coefficient satisfies $\left|r_{\mathrm{ss}0}\right|=0$ or $\left|r_{\mathrm{pp}0}\right|=-\left|r_{\mathrm{ss}0}\right|$ , the reflected transverse spatial displacement is effectively suppressed for *s*-polarized light. Notably, since *k* ^2^ *w* ^2^ is generally much larger than cot^2^ *θ*, equation (6) can be approximated as $\delta_{r\pm }^{s}\approx \mp \left(\left|r_{\mathrm{pp}0}\right|\left|r_{\mathrm{ss}0}\right|+{\left|r_{\mathrm{ss}0}\right|}^{2}\right)\cot\theta /\left(k{\left|r_{\mathrm{ss}0}\right|}^{2}\right)$ . Similarly, for $\left|r_{\mathrm{ss}0}\right|=0$ , both the numerator and denominator in the displacement expression become zero, resulting in a non-zero reflection transverse spatial displacement at the like-Brewster angle, as discussed in previous articles [6]. For $\left|r_{\mathrm{pp}0}\right|=-\left|r_{\mathrm{ss}0}\right|$ , this condition holds for arbitrary incident angles, wavelengths, and beam waists. In comparison with *p*-polarized light, we find that the condition $\left|r_{\mathrm{pp}0}\right|=-\left|r_{\mathrm{ss}0}\right|$ for the disappearance of reflection transverse spatial displacement applies to both pure *p*-polarized light and pure *s*-polarized light. When the reflection coefficient is complex at the interface, the condition is $\left|r_{\mathrm{ss}0}\right|=0$ or $\left|r_{\mathrm{ss}0}\right|=-\left|r_{\mathrm{pp}0}\right|\cos\left(\varphi_{r_{\mathrm{ss}0}}-\varphi_{r_{\mathrm{pp}0}}\right)$ for the disappearance of reflected transverse spatial displacement. There are analogous issues when $\left|r_{\mathrm{ss}0}\right|=0$ . For $\left|r_{\mathrm{ss}0}\right|=-\left|r_{\mathrm{pp}0}\right|\cos\left(\varphi_{r_{\mathrm{ss}0}}-\varphi_{r_{\mathrm{pp}0}}\right)$ , if $\varphi_{r_{\mathrm{ss}0}}=\varphi_{r_{\mathrm{pp}0}}$ , the condition for the disappearance of the reflected transverse spatial displacement becomes $\left|r_{\mathrm{pp}0}\right|=-\left|r_{\mathrm{ss}0}\right|$ ; and if $\varphi_{r_{\mathrm{ss}0}}=\varphi_{r_{\mathrm{pp}0}}\pm \pi$ , the condition turns into $\left|r_{\mathrm{pp}0}\right|=\left|r_{\mathrm{ss}0}\right|$ . Therefore, irrespective of the interface, the reflected transverse spatial displacement will vanish under the condition *r*_*pp*_ = −*r*_*ss*_ for an incident case of purely *s*-polarized light. This condition is consistent with the condition of equation (5).

Combining purely *p*-polarized light, it is plausible to conjecture that the reflected transverse spatial displacement will be effectively suppressed in equation (5), which is applicable to arbitrary linearly polarized light. By further substituting equations (5) into (3), we can obtain

(7) $$\delta_{r\pm }^{l}=0$$

These stipulations for the disappearance of displacement are also applicable to the first-order Taylor expansion of the Fresnel coefficient.

Upon analysis, three key points emerge. The initial point posits that the condition *r*_*pp*_ = 0 is pertinent solely to the incidence of pure *p*-polarized light, which effectively suppresses transverse spatial displacement. The second point suggests that *r*_*ss*_ = 0 serves to suppress the reflected transverse spatial displacement engendered by purely *s*-polarized light. It is imperative to note that both aforementioned points are subject to certain constraints, particularly in scenarios where *k* ^2^ *w* ^2^ ≫ cot^2^ *θ*. In contrast, the final point, which stipulates that *r*_*pp*_ = −*r*_*ss*_, represents a reflection condition that is efficacious for arbitrary polarization angles and incident angles, resulting in effective suppression of displacement. Note that the vicinity of *r*_*pp*_ = −*r*_*ss*_ does not bring a huge PSHE as near *r*_*pp*_ = 0.

## 3 Reflected transverse angular displacement

The reflected transverse angular displacement of left- and right-handed circular polarization components can be articulated as follows:

(8) $$\delta_{r\pm }^{l}=\mp \frac{z\cot\theta \left(L\mp M\right)}{n_{\text{out}}\left(C+D\pm F\right)}$$

where $L=2\cos2\beta \left[k^{2}w^{2}\left|r_{\mathrm{pp}0}\right|\left|r_{\mathrm{ss}0}\right|\sin\left(\varphi_{r_{\mathrm{ss}0}}-\varphi_{r_{\mathrm{pp}0}}\right)-\right.\left.\left|r_{\mathrm{ss}1}\right|\left|r_{\mathrm{pp}1}\right|\sin\left(\varphi_{r_{\mathrm{pp}1}}-\varphi_{r_{\mathrm{ss}1}}\right)\right]$ , and $M=\sin2\beta \left[k^{2}w^{2}\left({\left|r_{\mathrm{pp}0}\right|}^{2}-{\left|r_{\mathrm{ss}0}\right|}^{2}\right)-{\left|r_{\mathrm{ss}1}\right|}^{2}+{\left|r_{\mathrm{pp}1}\right|}^{2}\right]$ . In light of the non-zero displacement typically observed, the subsequent analysis shall focus on delineating the methodology for the eradication of the reflected transverse angular displacement.

For incident light that is purely *p*-polarized (transverse-magnetic, TM) or purely *s*-polarized (transverse-electric, TE), the reflected transverse angular displacement is distilled to its fundamental form

(9) $$\Delta_{r\pm }^{p}=\mp \frac{2z\cot\theta \left[k^{2}w^{2}\left|r_{\mathrm{pp}0}\right|\left|r_{\mathrm{ss}0}\right|\sin\left(\varphi_{r_{\mathrm{ss}0}}-\varphi_{r_{\mathrm{pp}0}}\right)-\left|r_{\mathrm{ss}1}\right|\left|r_{\mathrm{pp}1}\right|\sin\left(\varphi_{r_{\mathrm{pp}1}}-\varphi_{r_{\mathrm{ss}1}}\right)\right]}{n_{\text{out}}\left\{k^{2}w^{2}\left(k^{2}w^{2}{\left|r_{\mathrm{pp}0}\right|}^{2}+{\left|r_{\mathrm{pp}1}\right|}^{2}\right)+\left[k^{2}w^{2}J_{0}+J_{1}\right]\cot^{2}\theta \right\}}$$

(10) $$\Delta_{r\pm }^{s}=\pm \frac{2z\cot\theta \left[k^{2}w^{2}\left|r_{\mathrm{pp}0}\right|\left|r_{\mathrm{ss}0}\right|\sin\left(\varphi_{r_{\mathrm{ss}0}}-\varphi_{r_{\mathrm{pp}0}}\right)-\left|r_{\mathrm{ss}1}\right|\left|r_{\mathrm{pp}1}\right|\sin\left(\varphi_{r_{\mathrm{pp}1}}-\varphi_{r_{\mathrm{ss}1}}\right)\right]}{n_{\text{out}}\left\{k^{2}w^{2}\left(k^{2}w^{2}{\left|r_{\mathrm{ss}0}\right|}^{2}+{\left|r_{\mathrm{ss}1}\right|}^{2}\right)+\left[k^{2}w^{2}J_{0}+J_{1}\right]\cot^{2}\theta \right\}}$$

For simplicity, the Fresnel coefficients are not subjected to a Taylor expansion. When the reflection coefficient is purely real or imaginary, i.e., $\varphi_{r_{\mathrm{pp}0}}=\varphi_{r_{\mathrm{ss}0}}=0$ , the transverse angular displacements of the reflected light are effectively suppressed (i.e., $\Delta_{r\pm }^{p}=0$ and $\Delta y_{r\pm }^{s}=0$ ) for both *p*-polarized and *s*-polarized light. Analogous to transverse spatial displacement, if *k* ^2^ *w* ^2^ ≫ cot^2^ *θ*, equations (9) and (10) are simplified to $\Delta_{r\pm }^{p}\approx \mp 2z\left|r_{\mathrm{pp}0}\right|\left|r_{\mathrm{ss}0}\right|\sin\left(\varphi_{r_{\mathrm{ss}0}}-\varphi_{r_{\mathrm{pp}0}}\right)\cot\theta /\left(n_{\text{out}}k^{2}w^{2}{\left|r_{\mathrm{pp}0}\right|}^{2}\right)$ and $\Delta y_{r\pm }^{s}\approx \pm 2z\left|r_{\mathrm{pp}0}\right|\left|r_{\mathrm{ss}0}\right|\sin\left(\varphi_{r_{\mathrm{ss}0}}-\varphi_{r_{\mathrm{pp}0}}\right)\cot\theta /\left(n_{\text{out}}k^{2}w^{2}{\left|r_{\mathrm{ss}0}\right|}^{2}\right)$ , resulting in non-zero displacements at the Brewster angle for *p*-polarization and the like-Brewster angle for *s*-polarization. Should the reflection coefficient be complex, the angular displacement is reduced to $\Delta_{r\pm }^{p}=\mp 2z\left|r_{\mathrm{pp}0}\right|\left|r_{\mathrm{ss}0}\right|k^{2}w^{2}\sin\left(\varphi_{r_{\mathrm{ss}0}}-\varphi_{r_{\mathrm{pp}0}}\right)\cot\theta /\left\{n_{\text{out}}\left\{k^{2}w^{2}{\left|r_{\mathrm{pp}0}\right|}^{2}+\left[{\left|r_{\mathrm{pp}0}\right|}^{2}+{\left|r_{\mathrm{ss}0}\right|}^{2}+2\left|r_{\mathrm{pp}0}\right|\left|r_{\mathrm{ss}0}\right|\right.\right.\right.\left.\left.\left.\cos\left(\varphi_{r_{\mathrm{ss}0}}-\varphi_{r_{\mathrm{pp}0}}\right)\right]\cot^{2}\theta \right\}\right\}$ and $\Delta_{r\pm }^{s}=\pm 2z\left|r_{\mathrm{pp}0}\right|\left|r_{\mathrm{ss}0}\right|k^{2}w^{2}\sin\left(\varphi_{r_{\mathrm{ss}0}}-\varphi_{r_{\mathrm{pp}0}}\right)\cot\theta /\left\{n_{\text{out}}\left\{k^{2}w^{2}{\left|r_{\mathrm{ss}0}\right|}^{2}\right.\right.+\left.\left.\left[{\left|r_{\mathrm{pp}0}\right|}^{2}+{\left|r_{\mathrm{ss}0}\right|}^{2}+2\left|r_{\mathrm{pp}0}\right|\left|r_{\mathrm{ss}0}\right|\cos\left(\varphi_{r_{\mathrm{ss}0}}-\varphi_{r_{\mathrm{pp}0}}\right)\right]\text{co}{\text{t}}^{2}\theta \right\}\right\}$ for *p*- and *s*-polarized light beams, respectively. For both purely *p*- or *s*-polarized incident light, the displacement becomes zero when $\left|r_{\mathrm{pp}0}\right|=0$ or $\left|r_{\mathrm{ss}0}\right|=0$ or $\varphi_{r_{\mathrm{ss}0}}=\varphi_{r_{\mathrm{pp}0}}$ . Note that the same factors that contribute to the angular displacement not vanishing at the Brewster angle and the like-Brewster angle for the reflection condition of $\left|r_{\mathrm{pp}0}\right|=0$ or $\left|r_{\mathrm{ss}0}\right|=0$ also apply here. For the reflection condition of $\varphi_{r_{\mathrm{ss}0}}=\varphi_{r_{\mathrm{pp}0}}$ , both relationships *r*_*pp*_ = *r*_*ss*_ and *r*_*pp*_ = −*r*_*ss*_ are satisfied, thus causing the reflected transverse angular displacement to vanish at this juncture. Through analysis, it is determined that irrespective of the interface, the reflected transverse angular displacement $\Delta_{r\pm }^{l}=0$ will disappear in the cases where

(11) $$r_{pp}=r_{ss}\;\;\text{or}\;\;r_{pp}=-r_{ss}$$

which is applicable to arbitrarily linear polarized light and arbitrarily incident angles as per equation (8). The aforementioned conditions for the disappearance of angular displacement are also pertinent to the first-order Taylor expansion of the Fresnel coefficients.

Upon conducting the aforementioned analysis, it is postulated that when the Fresnel coefficient at the interface adheres to the condition *r*_*pp*_ = −*r*_*ss*_, the transverse spatial displacement of the reflected light is effectively suppressed, and concurrently, the transverse angular displacement of reflection is also eradicated. This phenomenon is graphically represented in Figure 1(a). Furthermore, linearly polarized light also adheres to this principle, as evidenced by equations (3) and (8). If the interface has a cross-reflection coefficient, this reflection condition inhibiting the displacement requires the addition of *r*_*ps*_ = *r*_*sp*_.

Given that conventional interfaces fail to meet the requisite reflection criteria, their construction is particularly significant. The tunable properties of two-dimensional materials offer a unique opportunity to achieve the aforementioned reflection conditions. By precisely controlling the electrical and magnetic surface conductivities [namely *σ*_*e*_ and *σ*_*m*_] of electromagnetic interfaces, it is possible to engineer interfaces capable of reflecting light with high precision. Regarding the design of the aforementioned interface, feasible solutions exist. For instance, reference [43] proposes a vertical heterostructure based on rule-based non-magnetic metasurfaces that aligns directly with optical interfaces possessing both magnetic and electrical surface conductivities. This approach has the potential to offer a universal platform for the flexible design of effective optical interfaces with on-demand magnetic surface conductivity.

## 4 Effectively suppressed reflected PSHE at the electromagnetic interface

Assuming the superstrate and substrate of the electromagnetic interface are identical, with relative permeabilities *μ*_*r*1_ = *μ*_*r*2_ = 1 and relative permittivities *ɛ*_*r*1_ = *ɛ*_*r*2_ = *ɛ*, we have rigorously solved for the reflection coefficients within the framework of macroscopic Maxwell’s equations, as stipulated by electromagnetic wave theory [44]. The resulting expressions are as follows:

(12) $$r_{pp}=1-\frac{\sigma_{e}}{\sigma_{e}+c\sqrt{\varepsilon }\varepsilon_{0}\cos\theta }+\frac{1}{c\sqrt{\varepsilon }\varepsilon_{0}\sigma_{m}\cos\theta -1}$$

(13) $$r_{ss}=\frac{c^{2}\varepsilon_{0}^{2}\varepsilon \sigma_{m}+\sigma_{e}\cos^{2}\theta }{\left(c\sqrt{\varepsilon }\varepsilon_{0}\sigma_{m}-\cos\theta \right)\left(c\sqrt{\varepsilon }\varepsilon_{0}+\sigma_{e}\cos\theta \right)}$$

where *ɛ*_0_ denotes the permittivity in vacuum, and *c* represents the speed of light.

**Figure 2:**
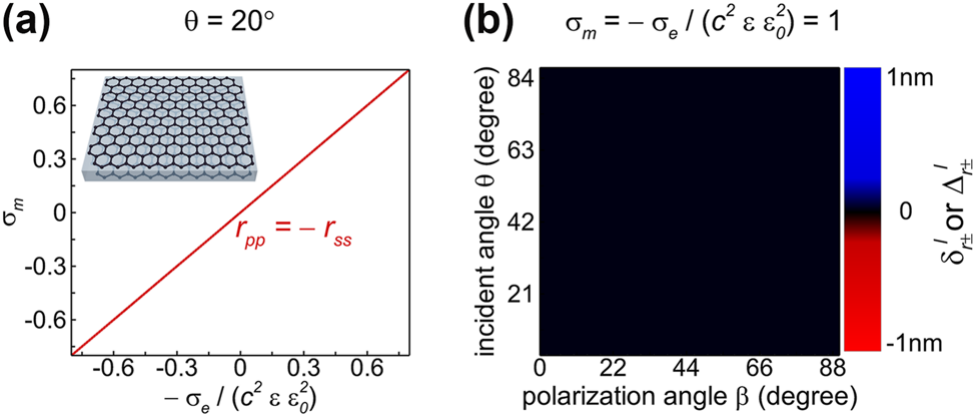
Polarization angle and incident angle influence on effectively suppressed PSHE of the reflected light at a designed electromagnetic interface with $\sigma_{e}=-c^{2}\varepsilon_{0}^{2}\varepsilon \sigma_{m}$ . (a) Draw a graph of *r*_*pp*_ = −*r*_*ss*_ based on the relationship between electric conductivity *σ*_*e*_ and magnetic conductivity *σ*_*m*_ when the incident angle takes *θ* = 20°. (b) Plot the transverse angular displacement *δ*_*r*±_ or angular displacement Δ_*r*±_ as a function of polarization angle *β* and incident angle *θ* at designed electromagnetic interface with $\sigma_{e}=-c^{2}\varepsilon_{0}^{2}\varepsilon \sigma_{m}=1$ .

Under this scenario, our engineered electromagnetic interface, characterized by the equation $\sigma_{e}=-c^{2}\varepsilon_{0}^{2}\varepsilon \sigma_{m}$ , meets the requisite reflection condition *r*_*pp*_ = −*r*_*ss*_. Figure 2(a) illustrates that the electric and magnetic surface conductivities adhere to the relationship $\sigma_{e}=-c^{2}\varepsilon_{0}^{2}\varepsilon \sigma_{m}$ , resulting in the reflection coefficients for *p*- and *s*-polarized light achieving *r*_*pp*_ = −*r*_*ss*_ at an incidence angle of *θ* = 20°. Consequently, in principle, the effectively suppressed reflected displacement $\delta_{r\pm }^{l}=\Delta_{r\pm }^{l}=0$ can be realized. As anticipated, when $\sigma_{e}=-c^{2}\varepsilon_{0}^{2}\varepsilon \sigma_{m}=1$ , both the reflected transverse spatial displacement $\delta_{r\pm }^{l}$ and the reflected transverse angular displacement $\Delta_{r\pm }^{l}$ remain consistently zero, irrespective of the incident angle *θ* and polarization angle *β*, as depicted in Figure 2(b). As a consequence, the effectively suppressed PSHE of the reflected light can be achieved at our engineered electromagnetic interface where *r*_*pp*_ = −*r*_*ss*_.

The effectively suppressed PSHE can be understood from the perspective of the phase. The reflection angular spectrum is reformulated as ${\bar{E}}_{r\pm }\approx \left[\left(r_{pp}+r_{ss}\right){\text{e}}^{\mp \text{i}2k_{ry}\cot\theta /k}{\text{e}}^{\pm \text{i}\beta }+\left(r_{pp}-r_{ss}\right){\text{e}}^{\mp \text{i}\beta }\right]\frac{w}{4\sqrt{\pi }}{\text{e}}^{-w^{2}\left(k_{ix}^{2}+k_{iy}^{2}\right)/4}$ . The coefficient in front of the index about the phase ∓2*k*_*ry*_ cot *θ*/*k* is *r*_*pp*_ + *r*_*ss*_. When *r*_*pp*_ = −*r*_*ss*_, this factor becomes zero, rendering the phase irrelevant. It explains the phenomenon that *r*_*pp*_ = −*r*_*ss*_ will lead to effectively suppressed PSHE of the reflected light, independent of the polarization status and the incident angle of the Gaussian beam. Of course, the effectively suppressed PSHE can also be explained from the perspective of spin–orbit interaction [41].

**Figure 3:**
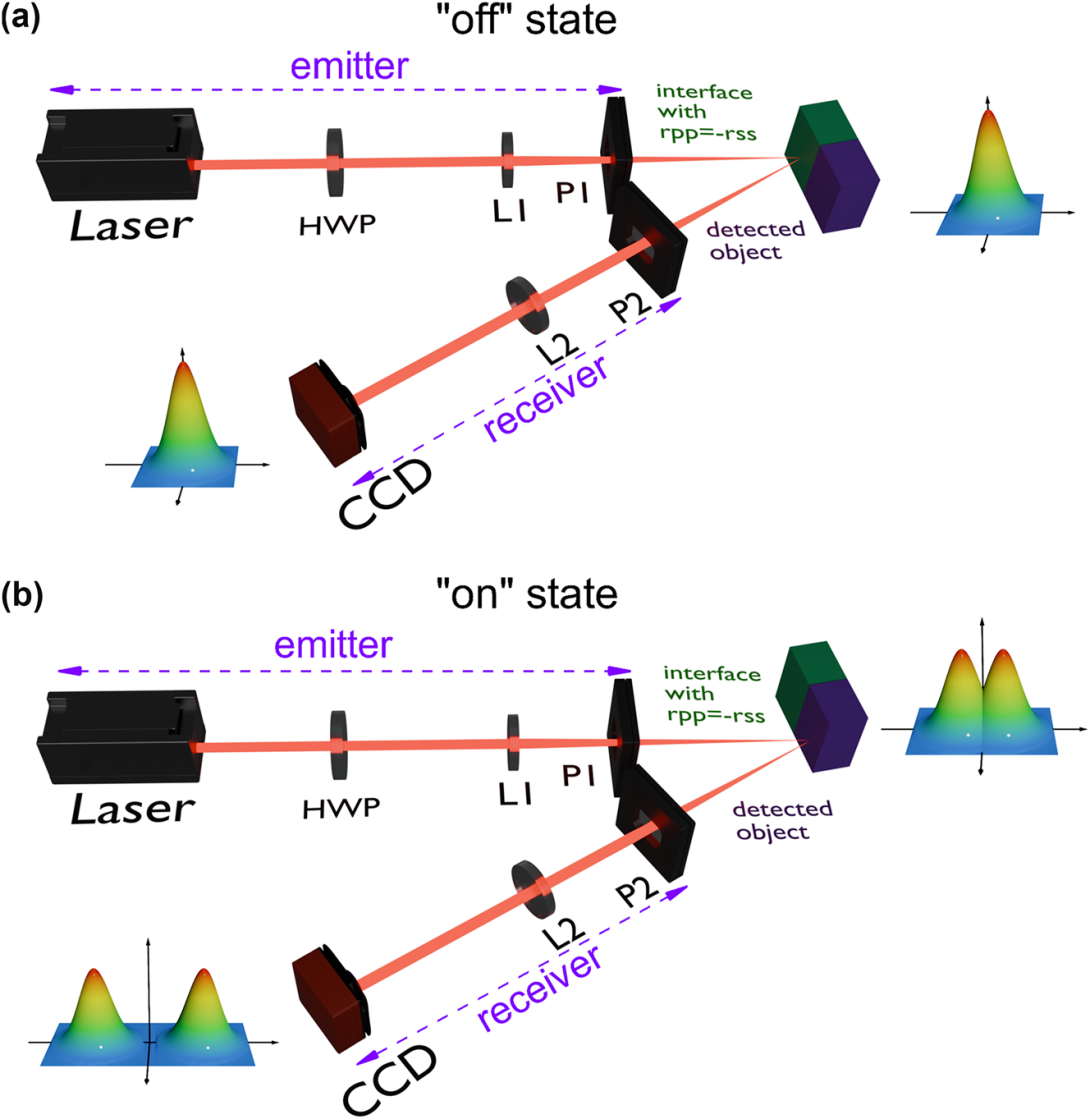
Schematic diagram of a spin switch mechanism predicated on the phenomenon of the reflected PSHE. (a) Depicted is the schematic of the optical switch in its “off” state, featuring a He–Ne laser as the light source, a half-wave plate (HWP) for polarization control, a lens (L1) for beam reduction, a polarizer (P1) for desired incident polarization state, an interface with *r*_*pp*_ = −*r*_*ss*_, another polarizer (P2) for post-selection, L2 and L1 forming a confocal cavity, and a charge-coupled device (CCD) for light intensity detection. The illustration on the right side of the interface shows the reflected light intensity in the absence of weak measurement, while the illustration to the left side of the CCD showcases the reflected light intensity subsequent to the implementation of weak measurement. (b) Illustrates the schematic of the optical switch in its “on” state.

The operational states of the reflective spin switch, predicated on the PSHE, are elucidated in Figure 3. The effectively suppressed PSHE constitutes the “off” state of the optical switch, as depicted in Figure 3(a). Conversely, the “on” state, which signifies the activated state of the optical switch, is portrayed in Figure 3(b). The foundational principle hinges upon the exploitation of spin and orbital angular momentum of light for information processing. This is accomplished by judicious selection of specific detection materials that facilitate the modulation and toggling of photonic signals. The He–Ne laser emits a beam of light, which passes through a device consisting of a half-wave plate (HWP), lens 1 (L1), and polarizer 1 (P1) to generate the incident beam required. This beam reflects off the interface and sequentially enters another polarizer (P2) selected for the purpose and another lens (L2) that forms a confocal cavity with L1, ultimately being received by a detector that captures the emitted optical signal. When the interface has *r*_*pp*_ = −*r*_*ss*_, the left-handed and right-handed components produced by reflection do not separate, as shown in the light intensity diagram on the right side of the interface in Figure 3(a). This phenomenon persists even after weak measurement protocols, as indicated by the corresponding light intensity diagram to the right of the CCD in Figure 3(a). This configuration signifies the “off” state of the reflective spin switch based on PSHE. However, a conventional interface does not typically conform to the condition of *r*_*pp*_ = −*r*_*ss*_, resulting in the divergence of the left-handed and right-handed components, as depicted in the light intensity diagrams before and after weak measurements in Figure 3(b). Consequently, for object detection, this scenario is analogous to the “on” state of the reflective spin switch based on the PSHE. The state of the switch can be ascertained by the segregation of light contingent upon its spin. Compared to traditional optical switches, this switch offers advantages such as high speed, high sensitivity, and the ability to handle multiple signals simultaneously. Given the rapid response characteristics of photons, the PSHE is particularly suitable for application in high-speed optical communication fields. Moreover, the PSHE is highly sensitive to physical parameters, and therefore, optical switches based on this effect can significantly enhance sensitivity during operation. It is worth noting that the left-handed and right-handed circular polarization components generated by the PSHE can achieve independent conduction of information due to their displacement characteristics. Traditional optical switches conventionally depend on the nonlinear optical or electro-optical properties of materials. In stark contrast, the switches predicated on the PSHE harness spin–orbit coupling to effectuate the spatial segregation of optical signals. This phenomenon thus provides novel concepts and mechanisms for the design and execution of optical switches. It is worth noting that the effectively suppressed reflected PSHE can be utilized not only for constructing spin switches but also for applications in quantum logic gates. By toggling the interface conditions (“on” or “off” states), it is possible to achieve the switching of photon spin states, and in the “off” state, it can also reduce crosstalk caused by spin splitting.

For the transmitted light beam, when *t*_*pp*_ = −*t*_*ss*_, the transmitted transverse spatial displacement can be represented as $\delta_{t\pm }^{l}=\pm kw^{2}\left(1+\eta \right)/\left[k^{2}w^{2}\tan\theta +{\left(1+\eta \right)}^{2}\right.\left.\cot\theta \right]$ , where *η* = cos *θ*_*t*_/cos *θ*. From the above equation, it can be observed that the transverse spatial displacement is generally not zero and does not depend on the polarization angle *β*. Therefore, unlike the reflected light beam, when *t*_*pp*_ = −*t*_*ss*_, the transmitted transverse spatial displacement is not effectively suppressed but becomes polarization-independent. Similarly, the transmitted angular displacement is always $\Delta_{t\pm }^{l}=0$ , indicating that the transmitted angular displacement is effectively suppressed.

## 5 Conclusions

In conclusion, we have effectively suppressed the reflected photonic spin Hall effect through a designed electromagnetic interface with necessary reflection conditions. Notably, the transverse spatial and angular displacements of the reflected light can always be zero, irrespective of the incident angle and polarization angle. Moreover, we have determined that the necessary reflection conditions for effectively suppressed photonic spin Hall effect can be achieved through the designed electromagnetic interface. This strategy effectively suppresses the spin splitting, thereby facilitating the “off” state of the spin switch. In addition, we have proposed a schematic representation of a reflective spin optical switch. This work provides novel insights and mechanisms for designing and implementing optical switches.
